# Astrocytes in Parkinson’s Disease: From Role to Possible Intervention

**DOI:** 10.3390/cells12192336

**Published:** 2023-09-22

**Authors:** Tianyou Wang, Yingqi Sun, Ulf Dettmer

**Affiliations:** 1 Collège Jean-de-Brébeuf, 3200 Chemin de la Côte-Sainte-Catherine, Montreal, QC H3T 1C1, Canada; 2 Department of Biochemistry, University of Oxford, Oxford OX1 3QU, UK; angela.sun@bioch.ox.ac.uk; 3 Ann Romney Center for Neurologic Diseases, Brigham and Women’s Hospital and Harvard Medical School, Boston, MA 02115, USA; udettmer@bwh.harvard.edu

**Keywords:** astrocyte, Parkinson’s disease, neuron–astrocyte interactions, glutamate, α-synuclein, disease-modifying therapy for PD

## Abstract

Parkinson’s disease (PD) is a neurodegenerative disorder characterized by the loss of dopaminergic neurons. While neuronal dysfunction is central to PD, astrocytes also play important roles, both positive and negative, and such roles have not yet been fully explored. This literature review serves to highlight these roles and how the properties of astrocytes can be used to increase neuron survivability. Astrocytes normally have protective functions, such as releasing neurotrophic factors, metabolizing glutamate, transferring healthy mitochondria to neurons, or maintaining the blood–brain barrier. However, in PD, astrocytes can become dysfunctional and contribute to neurotoxicity, e.g., via impaired glutamate metabolism or the release of inflammatory cytokines. Therefore, astrocytes represent a double-edged sword. Restoring healthy astrocyte function and increasing the beneficial effects of astrocytes represents a promising therapeutic approach. Strategies such as promoting neurotrophin release, preventing harmful astrocyte reactivity, or utilizing regional astrocyte diversity may help restore neuroprotection.

## 1. Introduction

Parkinson’s disease (PD) is the second most common neurodegenerative disease, second only to Alzheimer’s disease. It is also one of the world’s fastest-growing neurological disorders. In 2017, there were over one million patients in the US and the disease caused a significant economic burden (USD 51.9 billion) [1]. PD also has a severe impact on caregivers as well as the families of patients. Furthermore, its economic burden is only projected to increase, potentially surpassing USD 79 billion by 2037 [1]. Despite this severe socioeconomic burden, there is no definitive disease-altering treatment for PD since the available treatments, such as L-DOPA, only aim to improve symptoms. However, there are a few promising disease-altering drugs currently in clinical development [2]. One of PD’s distinguishing characteristics is the degeneration and the death of nigrostriatal dopaminergic (DAergic) neurons located in the midbrain substantia nigra pars compacta (SNpc), which invariably leads to symptoms such as bradykinesia, tremors, and rigidity [3]. These symptoms occur when the loss of SNpc neurons exceeds 70–80% [4]. Currently, the exact etiology of the disease is unknown. Nevertheless, the protein α-synuclein (αS) has been shown to play a major role in the development and pathogenesis of the disease. In its diseased state, αS was shown to form aggregates known as Lewy bodies, the major histological hallmark of PD [5]. Certain genetic mutations affecting αS production or its amino acid sequence, have been shown to cause familial PD or increase the risk of developing the disease. A wide range ofcellular mechanisms appears to be affected by PD, including neuroinflammation, faulty mitochondria, cellular damage due to oxidation, and the dysregulation of autophagy and mitophagy [6,7,8].

Astrocytes are the most common type of glial cells in the brain, which has an equal neuron–glia ratio [9,10]. Previously thought of as simply the “glue of the brain”, astrocytes are now increasingly recognized as performing numerous vital functions, for example, the maintenance of ion homeostasis and the blood–brain barrier. Astrocytes have also been shown to secrete neurotrophic substances, and certain genetic mutations associated with PD have been suggested to affect the function of astrocytes [11,12]. Although most research focuses on neuronal cells, the study of astrocytes should not be neglected due to their critical role in PD [11]. In this review, multiple aspects of astrocytes in PD will be discussed: the beneficial roles of astrocytes on neuronal survival, the causes of astrocyte dysfunction and its implications, and astrocyte therapy in PD. 

## 2. Beneficial Roles of Astrocytes to Neuronal Survival

**Figure 1:**
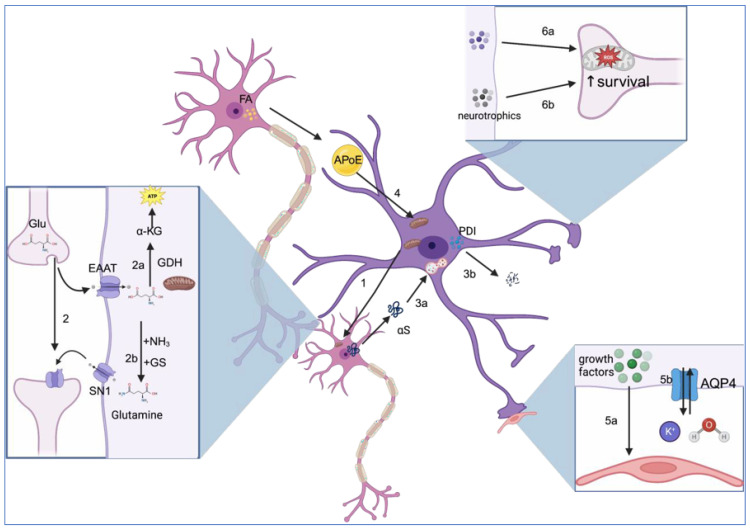
Astrocytes exhibit various neuroprotective properties. (1) Healthy astrocyte mitochondria are shown to be transferred to neurons during stress, which promotes a healthy neuron function. (2) Glutamate is taken up by astrocytes via EAAT. (2a) GDH, expressed in the mitochondria, converts glutamate into α-KG, a substrate for ATP production. (2b) With the usage of ammonia, the glutamine synthetase (GS) enzyme converts glutamate into glutamine, which is taken up by neurons via the SN1 transporters. (3a) Healthy astrocytes take up extracellular αS, which is degraded by the lysosome. (3b) PDI prevents extracellular αs fibrillization. (4) Fatty acids are transported from neurons to astrocytes by ApoE and converted into energy by astrocyte mitochondria. (5a) Growth factors secreted by perivascular astrocytes regulate and promote the secretion of tight junction proteins by endothelial cells, therefore maintaining the integrity of the BBB. (5b) AQP4 channels found in the end-feet of perivascular astrocytes regulate potassium ion and water homeostasis, implicating astrocyte activity in the function of the BBB. (6a) Molecules produced by astrocytes such as glutathione prevent oxidative stress in neurons. (6b) Astrocyte-secreted neurotrophic factors such as MANF promote neuron survival. Figure created with biorender.com. Accessed on 13 August 2023.

Astrocytes greatly contribute to neuronal survival via numerous mechanisms such as the secretion of neurotrophins and antioxidants, the removal of α-synuclein, glutamate metabolism, the metabolism of fatty acids, and the transfer of healthy mitochondria to neurons. However, it should be noted that the effects of astrocytes are not homogenous and depend on the localization of astrocytes in the brain [13]. For instance, reactive astrocytes are defined as astrocytes that have undergone various cellular, molecular, and functional changes in response to injury orneurodegenerative diseases [14]. Traditionally, they are classified as either A2 astrocytes that are considered beneficial to cell survival or harmful A1 pro-inflammatory astrocytes that do not exhibit the positive properties of A2 astrocytes [15]. However, these terminologies are oversimplified and do not consider the nuances of astrocytes [16]. As such, this paper, instead of using the terminologies A1 and A2, uses reactive astrocytes to refer to astrocytes in pathological conditions, whether positive or negative per the guidelines established by Escartin et al. [16]. The positive roles of astrocytes are discussed below and summarized in Figure 1.

### 2.1. Astrocytes Secrete Various Molecules That Are Beneficial to Neuronal Survival

In response to damage, reactive astrocytes have been shown to secrete a variety of neurotrophic factors (NTFs) [17]. While there are many secreted NTFs, the glial-cell-line-derived neurotrophic factor (GDNF) is the most studied and confers the most protection [18]. A study conducted by Lin et al., in 1994 demonstrated that this protein enhances the reuptake of dopamine by DAergic neurons via synapses and further enhances the survival of neurons [19,20]. Nevertheless, a certain degree of protection is also conferred by other NTFs such as mesencephalic astrocyte-derived neurotrophic factor (MANF) [12] or basic fibroblast growth factor [21].

Furthermore, astrocytes have been shown to be particularly important for protection against oxidative stress. Due to the high metabolic rate of the brain, 1–2% of oxygen in the mitochondria is converted into reactive oxygen species (ROS) instead of water [22]. Furthermore, an imbalance in the oxidant/antioxidant homeostasis leading to oxidative stress is implicated in the progression of several neurodegenerative diseases such as PD [23]. Hence, there are various biological processes that contribute to detoxifying ROS, one of which is the production of glutathione. While glutathione expression is not unique to astrocytes, astrocytes export 10% of their intracellular glutathione, making them special in this aspect, with the help of the multidrug resistance protein. This extracellular glutathione is provided to neurons, which helps protect them from oxidative stress. Furthermore, under oxidative conditions, astrocytes can export glutathione disulfide, another antioxidant that neutralizes ROS, thereby protecting the neurons [24].

Another potential pathway with which astrocytes promote neurogenesis and survival is possibly through the endocannabinoid system [25]. It has been demonstrated that the endocannabinoid 2-arachidonoylglycerol (2-AG) is neuroprotective through the receptor CB1R [26]. Astrocytes might be an important player in this as the deletion of diacylglycerol lipase alpha (DAGLA) in astrocytes, a gene producing a necessary enzyme for 2-AG production, negatively affected neuron survival and neurogenesis, suggesting that astrocytes play an important role in neurogenesis through the endocannabinoid pathway [25].

### 2.2. Removal of α-Synuclein 

α-Synuclein is a highly dynamic and generally intracellular protein that switches between structured and unstructured forms. In normal states, αS exists as monomers or multimers [27]. Some oligomeric, aggregated forms of αS are widely considered as toxic to neuronal cells [28]. However, the exact mechanisms behind the neurotoxicity of aggregated αS are not definitively known [29]. Furthermore, αS has been suggested to spread from neuron to neuron [30] via the initial release of αS, caused possibly in part by lysosomal exocytosis [31,32]. The extracellular αS would be taken up by other neurons, thus potentially infecting them [32]. Astrocytes also take in αS, but this process might be largely beneficial [33]. Indeed, astrocytes seem to have the capacity to intake extracellular αS and, through the process of phagocytosis, help to reduce neurotoxicity [33,34]. Extracellular αS can also be degraded by certain molecules released from astrocytes. Protein disulfide isomerase (PDI), a thiol-disulfide oxidoreductase, was shown to prevent extracellular αS fibrillization [35] and is found to be expressed in large quantities by astrocytes [33]. Nevertheless, evidence suggests that, under certain conditions, excessive αS could activate reactive astrocytes that adopt a pro-inflammatory phenotype that is detrimental to neuronal survival [36]. Furthermore, this extensive uptake causes the formation of intracellular deposits and mitochondrial damage [37].

### 2.3. Healthy Astrocyte Mitochondria Are Beneficial to Cellular Survival 

Healthy astrocyte mitochondria are shown to perform various functions that are beneficial to cellular survival, most notably we highlight the transfer of healthy mitochondria between neurons and astrocytes and the metabolism of fatty acids. These constitute essential functions for the survival of DAergic neurons. 

#### 2.3.1. The Transfer of Healthy Mitochondria between Neurons and Astrocytes Is Beneficial

A study by Hayakawa et al., in 2017 showed that astrocytes can transfer functional mitochondria to neurons. By using electron microscopy, the authors identified the presence of extracellular particles containing mitochondria from astrocytes originating from rats both in vivo and in vitro [38]. In the same study, cultures of astrocytes were grown and shown to release extracellular mitochondrial particles in a conditioned medium, particularly following the upregulation of CD38 in astrocytes [38], a protein likely to be involved in neurological crosstalk due to its increased expression during glutamate release from neurons [39]. The subsequent knockout of this protein inhibited astrocytic mitochondrial uptake by neurons and led to a poorer neuron survival outcome in vitro. These findings are supported by in vivo testing. Rat astrocytes were found to release extracellular mitochondrial particles in mice who had previously suffered from induced ischemia. Immunostaining revealed that these astrocytic mitochondria were present in neurons and led to increased ATP levels, demonstrating astrocytic mitochondria’s neuroprotective properties [38]. The poorer outcome associated with the inhibition of mitochondrial transfer due to CD38 KO further suggests that astrocyte mitochondria have a positive effect on neuron survival. This theory is also supported by other studies [40,41]. 

#### 2.3.2. Metabolism of Fatty Acids 

If in excess, fatty acids, in the form of neutral lipids such as triglycerides, are stored in lipid droplets (LDs) [42], which serve various functions. Notably, LDs transport fatty acids into mitochondria for energy consumption [43], avert excess cytosolic accumulation of fatty acids [44,45], and prevent fatty acids from undergoing lipid peroxidation, which generates toxic lipid peroxide [46]. They also stop fatty acids from being converted into acylcarnitines, which are known to cause mitochondrial damage [44]. A particularity with neurons is that the content of LD that they produce is typically very low [47]. Furthermore, their fatty acid consumption is limited since the associated processes generate oxidative species to which neurons are vulnerable [43]. As such, neurons must find a method to dispose of these fatty acids. In fact, the fatty acids of neurons are shown to be transported to astrocytes. Astrocytes represent a logical choice as they possess more adequate mechanisms to handle oxidative stress compared to neurons [48]. They also highly express genes involved in β-oxidation, a process that is necessary for the catabolism of fatty acids [49]. The fatty acids that are produced in hyperactive neurons are expelled to astrocytes with the help of the lipid transport protein ApoE (though a definitive mechanism remains to be elucidated) [47]. Thus, fatty acid metabolism by astrocytes might also protect neurons from excitotoxicity. Astrocytes possess N-methyl-D-aspartate (NMDA) receptors, which are glutamate receptors [50]. Ioannou et al., showed that glutamate decreases the number of LDs in astrocytes and theorize that this equates to the consumption of fatty acid and the production of ATP [47]. Glutamate is theorized to also trigger the release of ATP into the synaptic cleft [51], stimulating synaptic inhibition and inhibiting glutaminergic synapses via the activation of the P2X receptor, which leads to the inhibition of NMDA receptors [52,53]. Therefore, Ioannou et al., believe that the conversion of fatty acids into ATP could also be a method to prevent excitotoxicity in hyperactive neurons [47]. 

### 2.4. Excitotoxicity and Glutamate Metabolism

Glutamate is an excitatory neurotransmitter. Excess glutamate in the synaptic space is neurotoxic since it over-activates NMDA receptors, which leads to excess calcium inflow and triggers cell death signals. This eventually initiates neuronal necrosis and apoptosis [54].

Research has demonstrated that astrocytes can prevent these processes by playing a major role in glutamate uptake, removing 90% of glutamate in the CNS [55,56]. A study conducted by Rosenberg and Aizenman further explored the positive effects of astrocytes. When the growth of astrocytes was strictly controlled, cultures of rat cerebral cortexes were damaged even by the physiological levels of glutamate normally found in a rat’s hippocampus. However, when a sizeable quantity of astrocytes was added, the dosage of glutamate necessary to induce neurotoxicity was increased by 100 times [57]. This observation serves as a clear indicator of the neuroprotection roles that astrocytes provide by removing glutamate, which is achieved via various mechanisms. First, astrocytes express Na^+^-independent transporters [58], though the primary role of Na^+^-independent transporters is to uptake cystine. Nevertheless, it has been shown that astrocytes also uptake glutamate, albeit an extremely small quantity, through this channel. Moreover, evidence suggests that Na^+^-independent transporters uptake less than 5% of the total extracellular glutamate [59]. Astrocytes can also uptake glutamate via excitatory amino acid transporter (EAAT), which is responsible for most astrocyte glutamate intake since the knockout of EAAT2 was shown to increase glutamate levels [60]. Members of the EAAT transporter family transfer L-glutamate via the electrochemical gradient of K^+^ and Na^+^ [61,62]. While there are five members of the EAAT transporter family, only two are expressed in astrocytes (EAAT-1 and EAAT-2). 

After the uptake of glutamate, it is metabolized via two pathways. It is either converted to α-ketoglutarate (α-KG) or glutamine [63,64]. α-KG is a substrate used for the production of ATP via the Krebs cycle [63]. The complete breakdown of glutamate via oxidation is mainly accomplished through the process of oxidative deamination and is catalyzed by the enzyme glutamate dehydrogenase (GDH) [65]. Astrocyte mitochondria are essential to the enzymes in this pathway. This is because, in a rat model, GDH was found to be highly expressed in the mitochondria of astrocytes [66]. The other possibility that does not involve mitochondria is that glutamate is transformed into glutamine [64]. This transformation is caused by the cytosolic glutamine synthetase (GS) enzyme with the use of ammonia (NH3) [67]. This also prevents excess ammonia, which is neurotoxic [68]. Glutamine is then released from astrocytes via SN1 transporters and taken up by neurons [69], where it acts as a chemical precursor to numerous neurotransmitters such as GABA or glutamate [70,71,72]. This transformation from glutamine back to neurotransmitters also requires the presence of ammonium ions (NH^4+^), further regulating ammonia homeostasis [67]. In summary, astrocytes play a vital role in neuroprotection by taking up and metabolizing glutamate. 

### 2.5. Role in the Blood–Brain Barrier 

The blood–brain barrier (BBB) is the barrier between the brain and the blood that selectively allows certain molecules to pass through while restricting the access of others. Therefore, a functional BBB is integral to maintaining an optimal environment for the CNS. The BBB is regulated by a complex network, called the neurovascular unit (NVU), of astrocytes, endothelial cells, and specialized pericytes [73]. Healthy astrocytes play important roles in the maintenance of the BBB. Indeed, Hayashi et al., showed that cultures of endothelial cells possess better barrier functions and less permeability when grown with astrocytes [74]. The astrocyte-induced improvement in BBB function is caused by numerous factors. For example, via the secretion of growth factors, astrocytes can regulate the exocytosis of tight junction (TJ) proteins from endothelial cells [75]. Thus, endothelial–astrocyte communication also contributes to BBB integrity [75,76]. TJ proteins serve to inhibit the passage of molecules through the intercellular space and demarcate the apical and basolateral regions of the plasma membrane, thus facilitating endothelial cell polarity [77]. The regulation of BBB permeability is beneficial to the survival of neurons because selective impermeability prevents the infiltration of potential toxins. Astrocytes themselves might possibly secrete TJ proteins such as claudin-5 and occludin since astrocyte expression of occludin was observed. However, further research is required to confirm this idea because the detection of claudin-5 was not definitively found in the study [78]. Astrocytes also help the BBB to maintain water and potassium ion homeostasis. The end-feet of astrocytes, which cover the surface of capillaries, exercise many functions related to the BBB. The end-feet of perivascular astrocytes contain aquaporin channels (AQP) [79]. Human aquaporin channels can be divided into two types, AQP1 and AQP4, and the latter is highly expressed in astrocyte perivascular end-feet [79]. AQP4 channels regulate water homeostasis via bidirectional circulation, a vital function of the BBB [80]. Furthermore, AQP4 is potentially implicated in K^+^ homeostasis because a decrease in AQP4 expression is correlated with an alteration in K^+^ levels [81]. The antioxidants secreted by astrocytes, notably glutathione, might play a role in the maintenance of BBB permeability by reducing the capacity of molecules to penetrate into the brain, thus protecting the neurons from potential neurotoxins [82].

## 3. Neurotoxic Activities of Astrocytes

**Figure 2:**
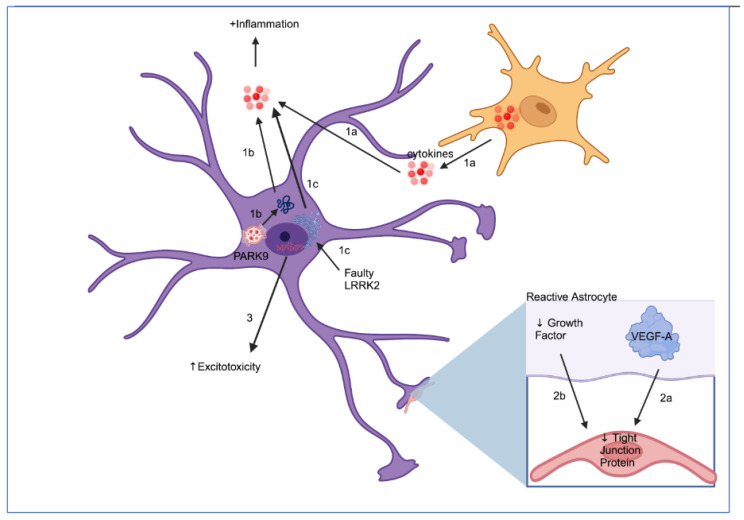
Various factors lead to astrocyte reactivity, which causes neurodegenerative properties in astrocytes. (1a) Microglia secrete various inflammatory cytokines that stimulate the expression of pro-inflammatory cytokines in astrocytes and turn them into a reactive phenotype. (1b) The loss-of-function mutation of PARK9 protein leads to an inability to neutralize αS, causing an accumulation of αS that induces astrocyte-mediated inflammation. (1c) LRRK2 mutation leads to ER stress-induced secretion of inflammatory cytokines through dyshomeostasis of calcium ions. (2a) Reactive astrocytes express VEGF-A, a protein that reduces BBB integrity by downregulating the expression of tight junction proteins, claudin-5 and occludin. (2b) Reactive astrocytes produce fewer growth factors, molecules implicated in tight junction expression, and thus BBB integrity. (3) Various genetic mutations found in PD such as LRRK2 and DJ-1 impair glutamate metabolism, which induces excitotoxicity in neurons. Figure created with biorender.com. Accessed on 13 August 2023.

While astrocytes have various neuroprotective activities that could potentially alleviate neuron death, PD seems to alter astrocyte function. Indeed, normal astrocyte functions such as glutamate metabolism and BBB regulation are affected in PD due to several factors. Additionally, the conversion of astrocytes into reactive pro-inflammatory astrocytes that release various inflammatory cytokines further worsens the conditions in PD. These negative aspects of astrocytes are explored below and are further summarized in Figure 2.

### 3.1. Glutamate Metabolism

As discussed above, astrocytes take up glutamate via transporters, thereby minimizing ecotoxicity. Various PD-linked genes have been suggested to affect this process, implying that alterations in astrocyte glutamate metabolism may play a role in PD. The leucine-rich repeat kinase 2 (LRRK2) gene, for example, has been proposed to regulate EAAT-2 expression. Certain mutations, such as the G2019S variant, have been shown to reduce EAAT-2 protein levels [83]. Due to the role of EAAT-2 in the regulation of glutamate levels [61,62], this would lead to excess glutamate and, invariably, excitotoxicity and DAergic neuron death. Similarly, DJ-1 mutations were also shown to impair EAAT-2 [84]. A deficiency in DJ-1, a protein implicated in PD that may associate with lipid rafts [85], has been shown to lead to a reduction in flotillin-1 and caveolin-1, both elements of lipid rafts [84]. Glutamate transporters, especially EAAT-2, may rely on lipid rafts to function [86]. Thus, glutamate-induced excitotoxicity will be elevated if lipid raft formation is impaired. Problems with glutamate metabolism in PD are also supported by the fact that glutamate uptake in platelets is reduced by 50% in PD patients compared to the control group [87]. Thus, glutamate uptake disruption may indeed be present in PD patients. This notion, coupled with studies supporting the disruption of EAAT transporters in astrocytes due to PD gene mutations [83,84], implicates the disruption of astrocyte glutamate metabolism in pathology, eventually leading to neuron death. 

### 3.2. Secretion of Inflammatory Cytokines

Inflammatory cytokines, such as TNF-α, IL-1β, and IL-6, appear to be implicated in PD since their levels are elevated in the disease [88,89,90]. Indeed, DAergic neurons appear to be susceptible to inflammatory cytokines. For instance, Aloe and Fiore found that TNF-α expression in the brains of mice limited tyrosine hydroxylase immunoreactivity in the caudate–putamen and affected grooming behavior [91]. The alteration of grooming behavior suggests that TNF-α affects neurons and the reduction in tyrosine hydroxylase immunoreactivity can be interpreted as a consequence of DAergic neuron loss induced by TNF-α [92]. A decrease n tyrosine hydroxylase corresponds to a decrease in dopamine levels, which underlies many of the symptoms seen in PD. 

Astrocytes are implicated in the production and release of inflammatory cytokines. The first pathway involves reactive microglia, which are present in sizeable quantities in PD [93]. Reactive microglia are shown to release various inflammatory cytokines in various diseases, such as IL-1α, leading to the conversion of astrocytes to a pro-inflammatory phenotype. Reactive neurotoxic astrocytes increase the expression of pro-inflammatory cytokines, amplifying neurotoxicity. Further exacerbating this problem is the fact that these astrocytes do not release neurotrophic factors or antioxidants [94]. 

However, other factors, unrelated to microglia, can induce inflammatory responses in astrocytes. While astrocytes have been shown to also possess the capacity to clear extracellular αS, αS aggregates will form inside astrocytes when degradation mechanisms are overwhelmed [95]. This accumulation was shown to induce various destructive properties of astrocytes such as inflammation [36,95]. The impairment of astrocyte lysosomes by bafilomycin A1 induced intracellular αS accumulation and increased the inflammatory response [95]. Consistent with this notion, various genetic mutations associated with PD seem to affect αS metabolism, which may not be restricted to neurons (the cell type with the highest endogenous levels of endogenous αS). Loss-of function mutation in PARK9, a gene associated with PD [96], causes impairments of lysosomes because PARK9 is necessary to maintain the acidic environment in the lysosome via the transportation of hydrogen and potassium ions [97]. If the lysosomal function in astrocytes is impaired, and, this leads to an accumulation of αS in astrocytes, thus causing astrocyte-induced inflammation [95]. Consequently, this affects extracellular αS levels. αS seems to also affect astrocytes by promoting endoplasmic reticulum (ER) stress in PD. The LRRK2 G2019S mutation, associated with PD, was suggested to increase astrocyte susceptibility to ER stress through the interaction with SERCA (a Ca^2+^ pump), leading to ER calcium depletion [98]. ER stress then can cause the secretion of inflammatory cytokines from astrocytes [98]. Another common genetic mutation in PD is in the beta-glucocerebrosidase gene (GBA-1). Mutations in this gene cause lysosomal defects, which in turn increase αS levels and possibly also causes the accumulation of glucosylceramide due to lysosomal defects [99,100,101]. While the exact consequence of genetic mutations in this gene in astrocytes remains to be elucidated, it has been demonstrated that GBA1 D409V alters the inflammatory response in astrocytes, which might be worthwhile to examine further [102]. DJ-1 mutations might also play a role in the modulation of neuroinflammation. The nuclear factor κΒ (NF-κΒ) has been shown to modulate inflammation and to promote neurodegeneration [103]. Indeed, the inhibition of NF-κΒ improved outcomes in spinal cord injuries [103]. While it is true that some research found that NF-κΒ is essential for the regulation of inflammation, imbalances to this signaling module exist in neurodegenerative diseases such as Alzheimer’s and aggravate neurotoxic astrocyte inflammation [104]. The deleterious effects of NF-κΒ activation are mitigated by the activation of nuclear factor erythroid 2-related factor (Nrf2), which promotes the release of antioxidative factors that counteract inflammation [105]. It has also been shown to downregulate microglia overactivation, something that causes astrocytes to adopt an inflammatory phenotype [93,106]. Nrf2 activation depends, however, on DJ-1, a gene that is altered in numerous familial cases of PD, suggesting that the Nrf2 pathway is affected [106]. Furthermore, NF-κΒ and Nrf2 are both expressed in astrocytes [107]. 

Moreover, astrocyte inflammasomes might play key roles in astrocyte-mediated inflammation. Pathological conditions result in an increase in lysophosphatidylcholine (LPC), generated through phospholipase A2 activity, which increases in neurological diseases [108,109]. LPC, a, damage-associated molecular pattern (DAMP), was shown to induce the activation of NLRC4 and NLRP3 inflammasomes in astrocytes that modulate cytokine IL-1β, and thus inflammation [109]. Even more, the NLRC4 inflammasome is highly regulated in astrocytes [109]. Nevertheless, not all inflammasomes activated by astrocytes are associated with cell death or inflammation and their function/effect is still not known. For instance, Barclay et al. found that the AIM2 inflammasome is activated in a rodent multiple sclerosis model at a late stage in astrocytes but not microglia [110]. However, AIM2 stimulation does not cause astrocytes to secrete a significant amount of IL-1β nor does it cause cell death, thus suggesting another function [110]. As such, the role of astrocyte-activated inflammasomes warrants further investigation.

### 3.3. Blood–Brain Barrier Disruption

Although previously not as extensively studied in PD, increasing evidence suggests that BBB dysfunction plays an active role in PD. BBB leakage is, interestingly, observed in PD patients in areas traditionally associated with PD, such as the basal ganglia [111]. This suggests that BBB disruption might be implicated in the development of PD. Since astrocytes are extremely vital for the BBB [112], astrocyte dysfunction might be a major cause of BBB disruption. As discussed in Section 2, astrocytes may promote the secretion of TJ proteins through the production of growth factors [75]. However, in PD, reactive astrocytes have been shown to significantly reduce their expression of growth factors [113] and thus, decrease the production of TJ proteins, leading to BBB leakage. Further exacerbating this issue, reactive astrocytes express VEGF-A, which downregulates tight junction proteins claudin-5 and occludin [114]. BBB leakage might contribute to neuron death by making the brain more susceptible to environmental toxins or other insults because the PD brain takes up drugs that would otherwise be blocked by the BBB [115]. 

## 4. Astrocyte Therapy in PD

The neurodegenerative and neuroprotective roles of astrocytes suggests that these cells might be a key piece of the puzzle in the elaboration of a disease-modifying treatment of PD. Indeed, astrocytes, as previously mentioned, have various positive effects such as the secretion of neurotrophic factors and the elimination of αS, and a key role in maintaining the equilibrium of the brain. However, as discussed in Section 3, certain reactive astrocytes are considered to have neurodegenerative effects. Thus, astrocytes should not be viewed as entirely positive, and this should not be overlooked in strategies involving astrocytes as part of a disease-modifying treatment. Therefore, a method, or a combination of methods, that would optimize the neuroprotective aspects of astrocytes but limit their neurodegenerative effects would be the most effective. Nevertheless, it should be noted that “astrocyte therapy” on its own is unlikely to definitively modify PD progression. Indeed, the full mechanisms of PD remain to be elucidated and all functions of astrocytes are still not fully understood or remain unknown. Additionally, αs is still likely to be the definitive player in the etiology of PD, which is considered a synucleinopathy [27]. Therefore, the best method might be to combine potential αS drugs, such as antisense oligonucleotides [116], with astrocyte therapy to optimize results; however, entirely halting αS production might be problematic. Current studies have shown that astrocytes can degrade at least a fraction of extracellular αS and prevent αS aggregation [33,34,35].

### 4.1. Promoting the Secretion of Neurotrophic Factors

Neurotrophic substances such as GDNF promote cellular survival. Therefore, artificially increasing the levels of these substances by injecting them into the bloodstream may offer certain benefits. Unfortunately, studies that have attempted to intravenously inject GDNF have failed [117,118], probably due to its difficulty in permeating the BBB to arrive at its targets. Furthermore, neurotrophins have a short half-life, which further complicates the development of an effective and realistic therapy. However, since astrocytes have been shown to naturally produce a variety of neurotrophins, as discussed in Section 2, a better way might be to develop a substance or method to stimulate or reactivate neurotrophin production. This would remove the need to find a way to allow neurotrophins, very large molecules, to traverse the BBB and might ensure a more even distribution of neurotrophins. Some authors have proposed the use of modified viruses expressing genes encoding neurotrophins [119,120]. Bäck et al., precisely tested this paradigm by injecting a modified adeno-associated virus encoding CDNF into rats’ striatum [119]. The results were mixed: CDNF was detected in the striatum and SN 12 weeks after injection, but there was no significant protection [119]. Similarly, in 2010, Marks et al., conducted a randomized controlled trial and found that the viral gene delivery for the trophic factor neurturin did not have any benefits [121]. Intriguingly, Cordero et al., found that the combination of CDNF and MANF overexpression by viral vectors conferred protection. This discrepancy in results is perhaps due to the fact that the dual overexpression of MANF and CDNF might confer more benefits than the expression of only one factor [122]. However, Cordero et al., used a 6-OHDA rat model [122], which is not a perfect representation of the etiology or development of PD compared to the controlled trial by Marks et al., involvingPD patients [121]. Furthermore, a possible reason may be the difference in the viral vector used, which might result in differences in gene expression or other diverging properties. Indeed, Cordero-Llana et al., used a lentivirus vector [122], while the two studies where the results were more modest used an adenovirus vector [119,121]. Therefore, since current studies seem contradictory, more research is needed on the best types of viral vector to use, as well as the efficacy of neurotrophin therapies. 

### 4.2. Prevention of Astrocyte Conversion into a Pro-Inflammatory Phenotype and Inflammatory Response 

Certain types of astrocytes secrete many inflammatory substances and are detrimental to neurons [94,123,124]. In PD, this conversion can be attributed to activated microglia [124]. A rational proposal would be to develop an inhibitor of this conversion. This seems to be a promising avenue since several preliminary studies discovered that certain molecules can inhibit reactive astrocyte activation. Capsaicin seems to be one of them, as discovered by Chung et al. [125]. Capsaicin, delivered through intraperitoneal injection, reduces the microglial expression of inflammatory cytokines, such as IL-1β in an MPTP rat model, through the TRPV1 receptors (capsaicin receptors) expressed in the brain [125]. Since inflammatory cytokines secreted by microglia activate reactive pro-inflammatory astrocytes [94], logically, pro-inflammatory astrocyte activation is reduced, as reported in [125]. However, these preliminary successes, while certainly encouraging, should be taken with a grain of salt. Simvastatin, an anticholesterol drug, was found to confer neuroprotection in neurotoxin models both in vivo and in vitro [126,127] in part due to its ability to prevent the conversion of astrocytes into a neurotoxic phenotype [126]. The results were encouraging, with Tong et al., reporting that an SH-SY5Y cell culture treated with simvastatin and 6-OHDA had a cell viability of 59.58 ± 5.80% in 24 h compared to only 47.34 ± 7.40% in SH- SY5Y cultures treated only with 6-OHDA; however, such success was not translated to success in clinical trials. In a 2022 randomized clinical trial involving 235 participants, Stevens et al., found that simvastatin was not effective as a disease-modifying drug and the drug takers had a worse performance in the MDS-UPDRS part 3 score by around 1.52 points in comparison to the control group [128]. The negative effect of simvastatin was supported by a 2017 case–control analysis showing that lipophilic statins, the group that simvastatin belongs to, are linked to an increased risk of PD [129]. There may, however, be an explanation for these seemingly conflicting data. The studies that found positive results were conducted either in in vitro tests, which did not completely simulate the complex dynamic of the real brain, or in in vivo tests, which used rat models induced with PD-like symptoms via neurotoxins [126,127]. Unfortunately, these models were shown to not be completely accurate as they did not completely model all PD parameters [130]. This discrepancy in results, along with that described in Section 4.1, further highlights the imperfection of models such as 6-OHDA. 

### 4.3. Astrocyte Graft

Astrocyte properties are not homogeneous throughout the entire brain and might vary significantly throughout regions [131]. Since PD mainly affects the neurons located in the substantia nigra, the introduction of astrocytes from different parts of the brain with different gene expression profiles that exhibit more neuroprotective behavior might help. One example is ventral midbrain (VM) astrocytes [33]. Yang et al., showed that this particular phenotype, when grafted into mouse brains, can inhibit intracellular and extracellular αS aggregation via numerous mechanisms. The transplantation of VM astrocytes can reduce αS accumulation and inflammatory cytokines in vivo while also restoring homeostasis of the brain [33]. This study convincingly showed the potential of utilizing astrocyte diversity. The benefits of astrocyte transplantation have also been confirmed in other studies in which cells differentiated into either astrocytes or astrocyte-like cells were beneficial once transplanted [132,133]. Indeed, after transplanting astrocyte-like cells into a 6-OHDA rat model, there was a marked enhancement in dopaminergic fiber density [133]. As our understanding of astrocyte heterogeneity grows, this method might become more appealing. While it is true that αS accumulation in astrocytes has been linked to a conversion into a more destructive phenotype [36,95], this property has not been observed in immature astrocytes [134]. This finding removes one potential risk of astrocyte graft: conversion into reactive pro-inflammatory astrocytes so long as the astrocytes are immature. Astrocyte graft can be combined with motor neurons to maximize its positive effects [33]. However, this technique is still rather experimental and certain aspects need to be improved before application. For instance, while it is true that, in the study conducted by Yang and colleagues, postnatal rodent astrocytes were utilized [33], there would be numerous ethical and logistical concerns about procuring human postnatal astrocytes on an industrial level. Therefore, human iPSCs should be prioritized. As the brain does not have full immune privilege contrary to what was previously theorized [135], there is still a certain response to the graft. This can be solved if the graft is autologous, but this technique, albeit more optimal compared with foreign iPSCs, is presently not as practical as in the case of DAergic neuron production [136]. Thus, further research is necessary. Furthermore, two other important questions to answer are whether there is a risk for glioma and what methods can be used to control excessive astrocyte proliferation. As of our current knowledge, no studies have been conducted on the risk of glioma in astrocyte grafts as most studies in this area involve rodents and not humans (difficulty translating results) and are not long-term. Moreover, while no excessive astrocyte proliferation has been reported in the scientific literature, this represents a question for this novel technique that must be answered for a successful clinical outcome. Since PD is clearly associated with a loss of DAergic neurons, it is logical to try to graft DAergic neurons as a way to replace the losses. However, certain data do not support the use of neuron grafting [137]. This may be due to the transfer of αS from the host to the graft, which stunts its potential effects [138]. However, since the astrocyte graft has been shown to prevent αS transmission, grafting neurons alongside astrocytes may prevent the neurons from developing αS aggregates, hence improving outcomes [33,139]. In summary, current evidence suggests that astrocyte grafts, particularly from certain parts of the brain, can reduce αS spread and may hold therapeutic value, especially when coupled with DAergic neuron grafts. 

### 4.4. Astrocyte Reprogramming

Since PD’s principal hallmark is a loss of DAergic neurons, replacing the dead neurons is a proposed solution. An exciting method to generate neurons is using microRNA (miRNA) to convert midbrain astrocytes into DAergic neurons [140]. This technique is particularly exciting as it is shown to work both in vivo and in vitro [141]. Indeed, Ghasemi-Kasman et al., converted astrocytes using a lentiviral particle carrying miR-302/367 and valproic acid with no detection of tumor [141]. Furthermore, this differentiation can be performed using other types of miRNAs (for a more detailed review of this, please refer to the review paper written by Wei and Shetty [140]). While this technique does appear to be promising, there are still some problems that need to be resolved. As astrocyte reprogramming does not resolve the αS problem, the new neurons will probably be infected with αS, as it is shown to spread [142]. Even more, as Wei and Shetty have noted, the newly reprogrammed neurons must be able to integrate into the existing neuronal network for positive results and in vivo programming might deplete astrocytes in the brain, thus limiting its efficiency [140].

## 5. Conclusions and Future Directions

In conclusion, this article highlights the nuanced role that astrocytes play in the brain and in PD, as well as their therapeutic potential. On the one hand, healthy astrocytes support neuronal survival through mechanisms such as releasing neurotrophic factors, clearing extracellular αS, metabolizing glutamate, transferring healthy mitochondria to neurons, and maintaining the BBB. On the other hand, dysfunctional astrocytes can drive disease progression. Disruption affects numerous aspects of vital astrocyte functions such as the clearance of αS, glutamate metabolism, and BBB maintenance, which invariably promote neurodegeneration. Targeting astrocytes represents a promising disease-modifying therapeutic approach for PD. Strategies such as promoting neurotrophin release, preventing pro-inflammatory astrocyte reactivity, and utilizing regional astrocyte diversity could restore neuroprotective functions. Combining astrocyte-based therapies with strategies that address αS pathology may provide synergistic benefits, as astrocyte therapy alone, based on the current understanding of astrocytes’ properties, seems unlikely to completely alter PD. However, more research is still needed to fully understand the nuances of astrocyte involvement in PD and to optimize astrocyte-targeted treatments. Translating findings from experimental models to PD patients remains an ongoing challenge. Accordingly, it may be necessary to exercise caution in interpreting any findings obtained using a neurotoxin. Answering these questions would help direct our limited resources in the right direction. To what extent do neurotoxin models replicate astrocyte reactions in real-life PD? To what extent does the lack of αS dysfunction and Lewy bodies in these models affect the accuracy of the results? Are animal transgenic/knockout models more accurate than neurotoxin models, or is a combination of both the best option? Key areas for future studies include further elucidating the molecular mechanisms of astrocyte dysfunction (and the extent to which astrocytes are affected) and develop a means to safely deliver effective therapeutic molecules or cells to affected brain regions and enhance the interaction between astrocytes and other glial cells, not just neurons. Even more, deep brain stimulation, a treatment commonly used in PD particularly when L-DOPA is ineffective, is not fully understood in all its facets and most probably has numerous mechanisms of action [143]. As astrocytes are very numerous in the brain, it would be interesting to examine the role of deep brain stimulation on astrocytes. Appreciating the pivotal and complex contributions of astrocytes will provide critical insights into tackling this devastating neurodegenerative disease.

## Data Availability

Not applicable.
